# The Metabolic Signature of Macrophage Responses

**DOI:** 10.3389/fimmu.2019.01462

**Published:** 2019-07-03

**Authors:** Antonella Viola, Fabio Munari, Ricardo Sánchez-Rodríguez, Tommaso Scolaro, Alessandra Castegna

**Affiliations:** ^1^Department of Biomedical Sciences, Istituto di Ricerca Pediatrica, University of Padova, Fondazione Città della Speranza, Padova, Italy; ^2^Department of Biosciences, Biotechnologies and Biopharmaceutics, University of Bari, Bari, Italy; ^3^IBIOM-CNR, Institute of Biomembranes, Bioenergetics and Molecular Biotechnologies, National Research Council, Bari, Italy

**Keywords:** macrophage, metabolism, inflammation, metabolic rewiring, immune cross-talk

## Abstract

Macrophages are a heterogeneous population of immune cells playing several and diverse functions in homeostatic and immune responses. The broad spectrum of macrophage functions depends on both heterogeneity and plasticity of these cells, which are highly specialized in sensing the microenvironment and modify their properties accordingly. Although it is clear that macrophage phenotypes are difficult to categorize and should be seen as plastic and adaptable, they can be simplified into two extremes: a pro-inflammatory (M1) and an anti-inflammatory/pro-resolving (M2) profile. Based on this definition, M1 macrophages are able to start and sustain inflammatory responses, secreting pro-inflammatory cytokines, activating endothelial cells, and inducing the recruitment of other immune cells into the inflamed tissue; on the other hand, M2 macrophages promote the resolution of inflammation, phagocytose apoptotic cells, drive collagen deposition, coordinate tissue integrity, and release anti-inflammatory mediators. Dramatic switches in cell metabolism accompany these phenotypic and functional changes of macrophages. In particular, M1 macrophages rely mainly on glycolysis and present two breaks on the TCA cycle that result in accumulation of itaconate (a microbicide compound) and succinate. Excess of succinate leads to Hypoxia Inducible Factor 1α (HIF1α) stabilization that, in turn, activates the transcription of glycolytic genes, thus sustaining the glycolytic metabolism of M1 macrophages. On the contrary, M2 cells are more dependent on oxidative phosphorylation (OXPHOS), their TCA cycle is intact and provides the substrates for the complexes of the electron transport chain (ETC). Moreover, pro- and anti-inflammatory macrophages are characterized by specific pathways that regulate the metabolism of lipids and amino acids and affect their responses. All these metabolic adaptations are functional to support macrophage activities as well as to sustain their polarization in specific contexts. The aim of this review is to discuss recent findings linking macrophage functions and metabolism.

## Introduction

From a historical perspective, macrophages (“*makros*” = big, “*phagein*” = to eat) were discovered in the 19th century by the Russian zoologist Metchnikoff, in a seminal study on starfish larvae. Metchnikoff observed that few hours after pinning them with small thorns of a tangerine tree, the thorns were surrounded by cells that he supposed to have origin from blood in response to injury (1). Macrophages were found in tissues as resident cells patrolling their surroundings and removing invading pathogens, apoptotic cells, and debris, thus maintaining tissue integrity. The first hypothesis was that tissue macrophages may differentiate from monocytes that exit the bloodstream during inflammation. However, it is now established that while monocyte-derived macrophages have origin in the bone marrow by definitive haematopoiesis, tissue macrophage progenitors derive from yolk sac and fetal liver, during primitive and definitive haematopoiesis (2). Interestingly, embryo-derived macrophages retain self-renewal potential, whereas monocyte-derived cells are terminally differentiated (3).

Despite of these differences, it is clear that both monocyte-derived and tissue-resident macrophages play a pivotal role in the maintenance of tissue homeostasis and in tissue regeneration after injury. In humans for example, the tissue cellular turnover rate has been estimated to be more or less 1 million cells per second each day (4): the removal of apoptotic cells is constantly provided mainly by macrophages that reside in tissues, through an immunologically silent process known as efferocytosis. One of the hallmarks of this process is represented by the release of anti-inflammatory cytokines that prevent the development of inflammation, such as interleukin (IL) 10 and transforming growth factor beta (TGF-β) (5, 6). Indeed, defects in the clearance of apoptotic cells are directly linked to the development of inflammation and autoimmune diseases (4).

On the other hand, when an inflammatory process is triggered by the perturbation of tissue homeostasis, bone-marrow derived monocytes that circulate in the blood-stream are attracted to the site of inflammation, through a specific milieu of pro-inflammatory chemokines secreted by resident macrophages, stromal and endothelial cells. At the site of inflammation, monocytes differentiate into macrophages, which cooperate with resident cells for sustaining immunity or promoting resolution of inflammation and tissue regeneration (7).

## The Two Poles of Macrophages Activation

Macrophages are extremely plastic cells being able to change rapidly their functional profile through a process defined as polarization. Macrophage polarization is indeed the process by which macrophages respond to stimuli coming from the local microenvironment and acquire a specific functional phenotype.

Based on specific programs of gene expression leading to the acquisition of different markers on the cellular surface, the secretion of certain cytokines as well as to metabolic adaptations, macrophages are usually classified into classically activated, pro-inflammatory or M1 macrophages (8, 9), and alternatively activated, anti-inflammatory, or M2 macrophages (10, 11). A classification of the different phenotypes is reported in Table 1.

**Table 1:** A schematic summary of macrophage polarization.

**Polarization**	**Stimuli**	**Released cytokines**	**Surface markers**	**Metabolic enzymes**	**Transcription factors**	**Functions**
M1	LPS + IFN-γ	TNF-α, IL-1β, IL-6, IL-12, IL-23	CD80, CD86, CIITA, MHC-II	iNOS, PFKFB3, PKM2, ACOD1	NF-κB (p65), STAT1, STAT3, IRF-4, HIF1α, AP1	Bacterial killing, tumor resistance, Th1 response
M2a	IL-4/IL-13	IL-10, TGF-β	CD206, CD36, IL1Ra, CD163	ARG1, CARKL	STAT6, GATA3, SOCS1, PPARγ	Anti-inflammatory response, tissue remodeling, wound healing
M2b	IC, TLR ligands/IL-1Ra	IL-10, IL-1β, IL-6, TNF- α	CD86, MHC II	ARG1, CARKL	STAT3, IRF4, NF-κB (p50)	Tumor progression, immunoregulation, Th2 response
M2c	Glucocorticoids/IL-10	IL-10, TGF- β	CD163, TLR1, TLR8	ARG1, GS	STAT3, STAT6, IRF4, NF-κB (p50)	Phagocytosis of apoptotic bodies, tissue remodeling, immunosuppresion
M2d (TAM)	TLR ligands + A2R/IL-6	IL-10, VEGF	CD206, CD204, CD163	ARG1, IDO	STAT1, IRF3, NF-κB (p50)	Angiogenesis, tumor progression

*A2R, adenosine receptor 2; ACOD1, aconitate decarboxylase 1; AP-1, Activator proten 1; ARG1, Arginase 1; CARKL, carbohydrate kinase- like; IC, immunocomplexes; IDO, indoleamine dioxygenase; iNOS, inducible Nitric Oxide Synthase; GATA3, GATA binding protein 3; GS, glutamine synthetase; HIF1α, Hypoxia-inducible factor 1-alpha; IFN-γ, Interferon gamma; IL-, interleukin; IRF, interferon regulatory factor; MHC-II, major histocompatibility complex class 2; NF-κB, nuclear factor kappa-light-chain-enhancer of activated B cells; PPARγ, Peroxisome proliferator-activated receptor gamma; SOCS1, Suppressor of cytokine signaling 1; STAT, Signal transducer and activator of transcription; TNF-α, Tumor necrosis factor alpha; TGF-β, transforming growth factor beta; TLR, toll like receptor; VEGF, Vascular endothelial growth factor*.

Pro-inflammatory macrophages are induced by microbial products, such as the lipopolysaccharide (LPS) and other Toll-like receptors (TLRs) ligands, or by cytokines secreted by T_H_-1 lymphocytes, such as interferon gamma (IFN-γ) and tumor necrosis factor alpha (TNF-α). From the functional point of view, M1 macrophages are characterized by their ability to kill pathogens and present their antigens to T lymphocytes for initiation of adaptive responses. Thus, they express CD80, CD86, CIITA, major histocompatibility complex class II receptor (MHC-II), cyclooxygenase 2 (COX-2), and inducible nitric oxide synthase (iNOS) and they produce high levels of pro-inflammatory cytokines, such as TNF-α, IL1-β, IL-6, IL-12, and IL-23, and promote T_H_-1 responses [extensively reviewed in (12, 13)]. The expression of these cytokines is mainly controlled by the activation and nuclear translocation of the transcription factor NF-κB (nuclear factor kappa-light-chain enhancer of B-cell) (14, 15), together with STAT1 (Signal transducer and activator of transcription) (16, 17), STAT3 (18), IRF4 (IFN-γ regulatory factor) (19), HIF1α (Hypoxia induced factor 1 alpha), and AP1 (activator protein 1) (20).

M2 or anti-inflammatory macrophages are induced by IL-4 or IL-13 secreted by innate and adaptive immune cells, such as mast cells, basophils, and T_H_-2 lymphocytes (10, 11). Alternatively-activated macrophages are characterized by an anti-inflammatory profile, which permits resolution of inflammation and tissue repair. They express high levels of mannose receptor (CD206), the decoy receptor IL-1R as well as the IL-1R antagonist, and produce pro-fibrotic factors such as the transforming growth factor beta (TGF-β) and insulin-like growth factor 1 (IGF-1), thus actively suppressing inflammation and promoting repair (21). In addition, markers and effectors associated with M2 polarization include STAT6, GATA3 (GATA binding protein 3), SOCS1 (suppressor of cytokine signaling 1), PPARγ (peroxisome proliferator-activated receptor gamma), CD163, CD36, FIZZ1 (found in inflammatory zone 1), matrix metalloproteases (MMPs), and arginase 1 (ARG1) (22). The increased arginase activity results in production of polyamines and collagen and favors tissue remodeling and wound healing (21). Finally, M2 macrophages induce angiogenesis and lymphangiogenesis by producing vascular endothelial growth factor A (VEGF-A), endothelial growth factor (EGF), platelet-derived growth factor (PDGF), and IL-8 (23).

In addition to this phenotype induced by IL-4/Il-13 (also known as M2a), specific profiles of M2 macrophages may be induced by different stimuli, including TGFβ, IL-10, immune complexes, or glucocorticoids (24). Thus, M2b or regulatory macrophages–induced by stimulation with immune complexes and TLR ligands or by IL-1R agonists–produce both pro- and anti-inflammatory cytokines, such as IL-10, IL-1β, and TNF-α, and regulate both immune and inflammatory reactions; on the other hand, the M2c subset is activated by glucocorticoids or IL-10 and exhibits a strong anti-inflammatory profile by releasing IL-10 and TGF-β. Finally, M2d macrophages, also known as tumor-associated macrophages (TAMs), are induced by TLR ligands and A2 adenosine receptor (A2R) agonists, or by IL-6; they secrete high levels of IL-10, TGF-β, and VEGF and low IL-12, TNF-α, and IL-1β, and contribute to tumor angiogenesis, growth and metastasis (25).

Considering the complexity of the tissue microenvironment and the plasticity of macrophages, it is clear that a static vision of M1–M2 polarization adopted from *in vitro* experiments may not fully describe macrophage polarization *in vivo*, which has to be considered as an extremely dynamic and tissue-specific process.

## Macrophage Metabolism

**Figure 1:**
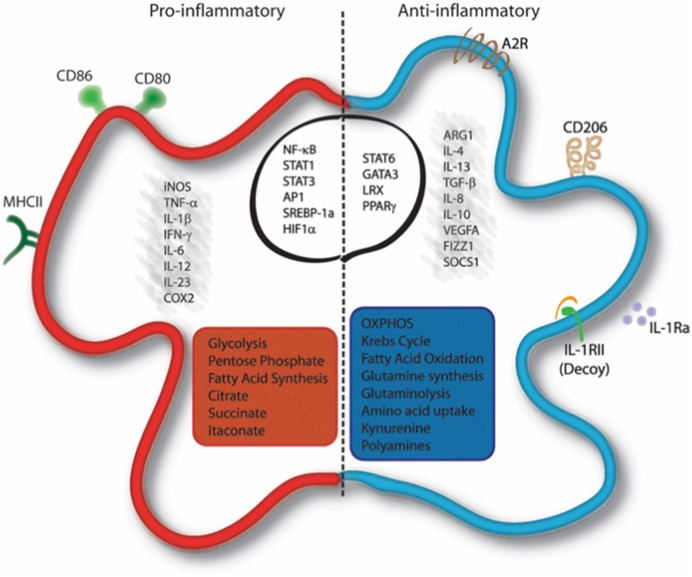
Molecular and metabolic signatures of macrophage activation. Pro-inflammatory stimuli induce the activation of specific pathways through the activation of transcription factors such as NF-κB, STAT1, STAT3, AP-1, SREBP-1, and HIF1α, which trigger the expression of markers like iNOS, COX-2, CD80, CD86, and MHC-II and the release of IL-1β, TNF-α, IFN-γ, IL-6, IL-12, and IL-23. Cells undergo a metabolic reprogramming toward glycolysis, the pentose-phosphate pathway, and fatty acid synthesis. This associates to interruption of the Krebs cycle, ROS formation and efflux of citrate, which supports NADPH and PGE2 synthesis, and succinate, which stabilizes HIF-1α. Itaconate is produced from citrate and displays antibacterial function. Anti-inflammatory macrophages are characterized by the expression of ARG1, FIZZ1, SOCS1, CD206, Adenosine receptor (A2R), and the decoy IL1RII and by the production of cytokines such as TGF-β, IL-10, IL-4, IL-13, IL-8, IL-1Ra, and VEGFA. Their profile is mainly controlled by the activity of the transcription factors STAT6, GATA3, PPARγ, and LRX. Metabolically, these cells display enhanced OXPHOS metabolism, fatty acid oxidation, glutaminolysis, tryptophan catabolism with release of kynurenine, and synthesis of polyamines. AP-1, Activator proten 1; ARG1, Arginase 1; COX2, cicloxygenase 2; FIZZ1, Found in inflammatory zone 1; iNOS, inducible Nitric Oxide Synthase; GATA3, GATA binding protein 3; HIF1α, Hypoxia-inducible factor 1-alpha; IFN-γ, Interferon gamma; LXR, Liver X receptor; MHC-II, major histocompatibility complex class 2; NF-κB, nuclear factor kappa-light-chain-enhancer of activated B cells; PPARγ, Peroxisome proliferator-activated receptor gamma; SOCS1, Suppressor of cytokine signaling 1; SREBP-1, Sterol regulatory element binding protein 1; STAT, Signal transducer and activator of transcription; TNF-α, Tumor necrosis factor alpha; TGF-β, transforming growth factor beta; VEGFA, Vascular endothelial growth factor A.

In addition to the functional properties mentioned above, macrophage polarization involves also metabolic reprogramming (Figure 1). Thus, depending on the stimuli received by the microenvironment, macrophages can switch from an aerobic profile, based on oxidative phosphorylation, to an anaerobic one, based on glycolysis, and vice versa.

The first studies in the field of immune cell metabolism appeared in the 1950s, with the discovery that neutrophils depend on aerobic glycolysis, a process defined as “Warburg effect” (26). Indeed, this metabolic pathway was first recognized by Otto Warburg, during his research on tumor cells, which are characterized by increased glucose uptake, high rate of glycolysis, followed by lactic acid fermentation in conjunction with a reduced level of oxidative phosphorylation (OXPHOS), even in the presence of abundant oxygen. In this setting, aerobic glycolysis occurs to produce energy and to generate biosynthetic intermediates (26, 27). In 1970, Hard et al. observed that M1 macrophages display enhanced glycolysis accompanied by decreased oxygen consumption (28, 29). Almost 20 years later, Newsholme et al. demonstrated that the rate of glycolysis increased dramatically during phagocytosis or upon macrophage activation by inflammatory stimuli. Indeed, now we know that pro-inflammatory macrophages utilize glycolysis (29, 30) and the pentose phosphate pathway (PPP) (31, 32) to meet their ATP requirements, whereas the Krebs cycle is broken at two points (32, 33), and OXPHOS as well as the fatty acid oxidation (FAO) are downregulated (32, 34, 35). In contrast, in M2 macrophages the Krebs cycle is intact and their metabolic activity is characterized by enhanced FAO and OXPHOS (32).

Starting from these and other observations, the concept of “immunometabolism” has been introduced to indicate that, in addition to provide energy supporting immune activity in specific contexts, these metabolic adaptations directly affect immune cell functions by controlling transcriptional and post-transcriptional events. In the next paragraph we will describe the main metabolic blocks and the modulation of their fluxes for sustaining the different functional states of macrophages.

## Glycolysis and the Pentose Phosphate Pathway (PPP)

**Figure 2:**
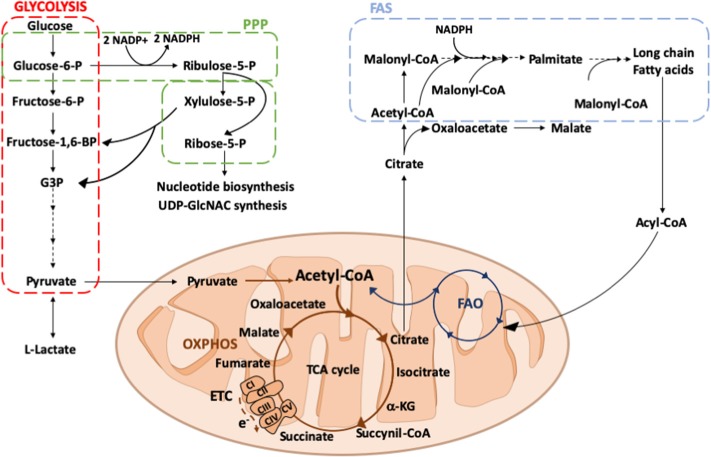
Overview of glucose and fatty acid metabolism. Glucose is converted into pyruvate by glycolysis (red square), in the cytosol. Among the glycolytic intermediates, glucose-6P can be diverted into PPP (green square) sustaining NADPH and ribose-5P production that, in turn, are used for fatty acid or nucleotide and UDP-GlcNAC synthesis, respectively. In hypoxic conditions, pyruvate is preferentially reduced to lactate, whereas in normoxic conditions it is decarboxylated into acetyl-CoA within the mitochondria. Here, acetyl-CoA enters into the TCA cycle, providing reducing agents to the ETC to generate energy. Citrate, an intermediate of the TCA cycle, can be exported into the cytosol where it participates in fatty acid synthesis (FAS; light blue square). Fatty acids can be oxidized via FAO (dark blue square) within the mitochondrial matrix thus generating acetyl-CoA to replenish the TCA cycle. PPP, pentose phosphate pathway; ETC, electron transport chain; TCA, tricarboxylic acid; OXPHOS, oxidative phosphorylation; FAS, fatty acid synthesis; FAO, fatty acid oxidation; Glucose 6-P, glucose 6-phosphate; Fructose 6-P, fructose 6-phosphate; Fructose-1,6-BP, fructose 1-6-biphosphate; G3P, glyceraldeyde 3-phospate; Acetyl-CoA, acetyl-Coenzyme A; α-KG, alpha-ketoglutarate; e-, electrons; CI, CII, CIII, CIV, CV, complex I, II, III, IV, V; UDP-GlcNAC, Uridine diphosphate N-acetylglucosamine.

Glycolysis is one of the simplest ways to generate energy within the cell (Figure 2). The glycolytic metabolic pathway takes place in the cytosol and it converts glucose to pyruvate, thus generating two molecules of ATP per unit of glucose. Although glycolysis is relatively inefficient in ATP production, it provides metabolic intermediates for biosynthetic pathways to support the synthesis of ribose, amino acids, and fatty acids that are crucial for metabolic adaptation of the cell. Furthermore, glycolysis supplies the PPP, allowing the production of NADPH and ribose-5-phosphate. In parallel to glycolysis, PPP occurs in the cytosol and consists of two distinct phases. In the oxidative phase, the energy from metabolic conversion of glucose-6-phosphate into ribulose-5-phosphate is used for the reduction of NADP^+^ into NADPH. NADPH is then used by several enzymes, including the NADPH oxidase, which generates reactive oxygen species (ROS) to kill pathogens and plays a crucial role in macrophage responses (36, 37). Moreover, high levels of NADPH offer protection against oxidative stress, by providing reducing power for generation of the antioxidant glutathione (38). In the non-oxidative phase, intermediates from glycolysis are diverted for the synthesis of ribose-5-phosphate, a precursor of nucleotides, and amino acids.

Glycolysis is a crucial metabolic event for M1 macrophages and its inhibition affects many functions typical of their inflammatory phenotype, including phagocytosis, ROS production, and secretion of pro-inflammatory cytokines (29, 39, 40). Glycolytic metabolic adaptation relies on the activation of several transcription factors, among which HIF1α plays a key role in the commitment to glycolysis also under normoxic conditions (41).

In macrophages, two main signaling pathways culminate in oxygen-independent regulation of HIF1α transcription: the TLR/NF-κB (42) and AKT/mTOR (43–45) pathways. Several inflammatory signals, such as pathogen recognition through pattern recognition receptors (PRRs) or pro-inflammatory cytokines, converge in NF-κB activation, the master regulator of macrophage functions that regulates the expression of several genes, including HIF1α (46, 47). On the other hand, the AKT/mTOR pathway is triggered by growth factors, such as GM-CSF, and pathogen-sensing receptors, such as Dectin-1 or TLR4 (45, 48, 49). Interestingly, mTORC1 also increases the expression of genes involved in mitochondria biogenesis and oxidative metabolism, such as PPAR-γ and Yin Yang 1 (YY-1) (50). On this line, Akt kinases seem to regulate macrophage polarization in an isoform-specific manner: while Akt1 deletion promotes the M1 profile, deletion of Akt2 has opposite effects, resulting in amplification of M2 responses (44). In addition to these signaling pathways, in M1 cells HIF1α expression may also be stabilized by succinate coming from the TCA breakpoint at succinate dehydrogenase (SDH) (31) (see “Krebs cycle” section).

In macrophages, HIF1α acts as a metabolic and functional regulator of cell responses, regulating the expression of genes encoding for glycolytic enzymes, the glucose transporter GLUT1, as well as inflammatory mediators (41, 42, 47). The upregulation of GLUT1 is important for the glycolytic activity of M1 macrophages as it facilitates rapid glucose uptake (29). Additionally, HIF1α supports the conversion of pyruvate into lactate by promoting the expression of two enzymes: the lactate dehydrogenase (51), which produces lactate from pyruvate, and the pyruvate dehydrogenase kinase (52, 53), which inactivates pyruvate dehydrogenase thus limiting pyruvate entering into the Krebs cycle. In M1 macrophages, in which OXPHOS is limited, the conversion of pyruvate into lactate is essential to restore NAD^+^ and maintain flux through the glycolytic pathway.

Two additional points of the glycolytic flux regulation occur at the level of the 6-phosphofructo-2-kinase B (PFKFB) and the pyruvate kinase M2 (PKM2). M1 macrophages express predominantly the PFKFB3 isoform (53) which, if compared to the other isoforms, less efficiently catalyzes the conversion of fructose-2,6-bisphosphate in fructose 6-phosphate, enhancing the glycolytic flux. Moreover, M1 cells upregulate the isoform 2 of the pyruvate kinase (PKM2), which plays multiple roles in macrophage metabolism and polarization. Indeed, when highly expressed, PKM2 exists in an equilibrium of enzymatically inactive monomers or dimers and enzymatically active tetramers (53). The inactive enzyme translocates into the nucleus and, by binding to HIF1α, triggers the expression of HIF1α-regulated genes (53–56), whereas the enzymatically active tetramers are retained in the cytoplasm and promote glycolysis as well as M1 polarization (53).

As mentioned above, the oxidative steps of the PPP are crucial for macrophages: oxidation of glucose leads to the reduction of NADP^+^ to NADPH, which is fundamental not only for NADPH oxidase and macrophage's killing activity but also for anti-oxidant defense mechanisms and fatty acid biosynthesis, required for prostaglandin production. Indeed, the oxidative PPP activity is prominent in M1 macrophages (31) and the knockdown of 6-phosphogluconate dehydrogenase (PGD), which converts 6-phosphogluconate into ribulose 5-P, generates a deficient pro-inflammatory response in macrophages during hypercholesterolemia (57). On the other hand, as expected, the non-oxidative branch of PPP is repressed in M1 macrophages (32). This occurs through the downregulation of sedoheptulose kinase (CARKL), a carbohydrate kinase-like protein that is involved in the conversion of sedoheptulose into sedoheptulose-7-phosphate (58). In line with this finding, overexpression of CARKL in macrophages results in defective M1 polarization and dampened inflammatory response (57, 58).

The role of glycolysis in M2 macrophage functions is more controversial. Several studies have shown that glycolysis is active in M2 cells and that its blockade with 2-deoxyglucose (2-DG), a well-established glycolysis inhibitor, may inhibit M2 polarization and functions (19, 59). On the other hand, more recent data suggest that glycolysis is not required for M2 differentiation, as long as OXPHOS remains intact (60). This suggests that M2 macrophages display a more flexible metabolic activity since they can supply OXPHOS even in absence of glycolysis using glutamine (60). Another control point of glycolysis in M2 macrophages is represented by the selective expression of the glycolytic enzyme 6-phosphofructo-2-kinase B1 (PFKFB1), which much more efficiently catabolizes fructose-2,6-bisphosphate, an activator of glycolysis, to fructose-6-phosphate, lowering the glycolytic rate (61, 62). Finally, in the M2 phenotype, CARKL is upregulated, enhancing the non-oxidative steps of PPP, which can lead to ribose-5P production, necessary for nucleotide and UDP-GlcNAC synthesis (58). UDP-GlcNAC is required for N-glycosylation, which is essential for the modification of different cell surface proteins (i.e., CD206) abundantly expressed in M2 macrophages (32).

The metabolic differences between M1 and M2 cells impact on the ability of these cells to generate ROS. In a situation of coupled and efficient respiration, the amount of ROS produced by the electron transport chain (ETC) is kept under control and at low levels. In conditions of OXPHOS dysfunction, a significant leakage of electrons occurs, which, in the presence of oxygen, produces ROS (63, 64). This is the case of pro-inflammatory macrophages, in which polarization profoundly modifies the OXPHOS, leading to ROS production. Although CI and CIII are considered the main sites of mitochondrial ROS production, recent studies suggest that ROS are generated by reverse electron transport (RET) at CI of the ETC rather than CIII, in a situation of impaired OXPHOS (65). The evolutionary conserved signaling intermediate in Toll pathways (ECSIT), a TRAF6 target for ubiquitination and CI-associated protein, is a master regulator of ROS production and mitochondrial quality control in macrophages. In particular, after phagocytosis of bacteria, ECSIT triggers recruitment of mitochondria to the phagosome to produce ROS that activate NADPH oxidase to kill bacteria (63). Other than being a harmful byproduct of metabolism, cellular ROS have emerged as master regulators of cellular signaling through the activation of many redox-sensitive pathways [extensively reviewed in (66)]. In macrophages, ROS are known to regulate several functions, including phagocytosis, bacterial killing, and polarization into specific phenotypes. Mitochondrial ROS are known to sustain inflammation by mediating IL-6, TNF-α, and IL-1β cytokine secretion, through a mechanism involving mitochondrial ROS-dependent MAPK activation (67).

## The Krebs Cycle

Once pyruvate is generated, it becomes oxidized through a series of reactions termed the Krebs or Tricarboxylic Acid (TCA) cycle (Figure 2). The continuous flux through this cycle utilizes acetyl CoA, deriving from the breakdown of carbon-based nutrients, to reduce NAD^+^ and FAD to NADH and FADH_2_, which are then oxidized leading to ATP production. M2-like macrophages are known to display a functional and intact TCA cycle, which is crucial to meet the ATP demand due to the high (UDP-GlcNAc requiring) glycosylation levels of lectin and mannose receptors necessary for M2 macrophage function (32). At variance with M2 metabolism, the increased flux of glycolysis displayed by M1 macrophages is accompanied by metabolic changes involving the Krebs cycle, which are not only important for anabolic or energetic purposes, but also for sustaining the inflammatory response (68–70). Indeed, in pro-inflammatory macrophages, the Krebs cycle is interrupted at several key points allowing signal metabolites citrate, succinate and itaconate to escape mitochondria and exert their regulatory role.

### Citrate

Citrate production and conversion connects mitochondrial and cytosolic metabolism. It is produced in the Krebs cycle by condensation of oxaloacetate and acetyl-CoA, the latter deriving from glycolytic pyruvate or from the catabolism of fatty acids. Citrate is converted to isocitrate and then to α-ketoglutarate (αKG), through the activity of isocitrate dehydrogenase (IDH). However, citrate can also be exported into the cytosol in exchange with malate through the transport activity of the mitochondrial citrate carrier (CIC), also known as solute carrier family 25 member 1 (SLC25A1) (71, 72). Once in the cytosol, citrate displays a plethora of regulatory roles. It inhibits glycolysis, by acting directly on phosphofructokinase (PFK) 1 and 2 and, indirectly, on pyruvate kinase (PK) (73); it stimulates lipid synthesis, through the activation of acetyl-CoA carboxylase (ACC) (74), and gluconeogenesis, through the activation of fructose-1,6-bisphosphatase. Cytosolic citrate is also a substrate of ATP-Citrate lyase (ACLY), producing acetyl-CoA and oxaloacetate (72). Oxaloacetate can be converted to malate by malate dehydrogenase (MDH). Malate can be transported back into the mitochondrial matrix in exchange with citrate through CIC (75) or can lead to pyruvate through the NADPH producing-malic enzyme (76). Through ACLY activity, cytosolic citrate positively regulates protein and histone acetylation (77).

**Figure 3:**
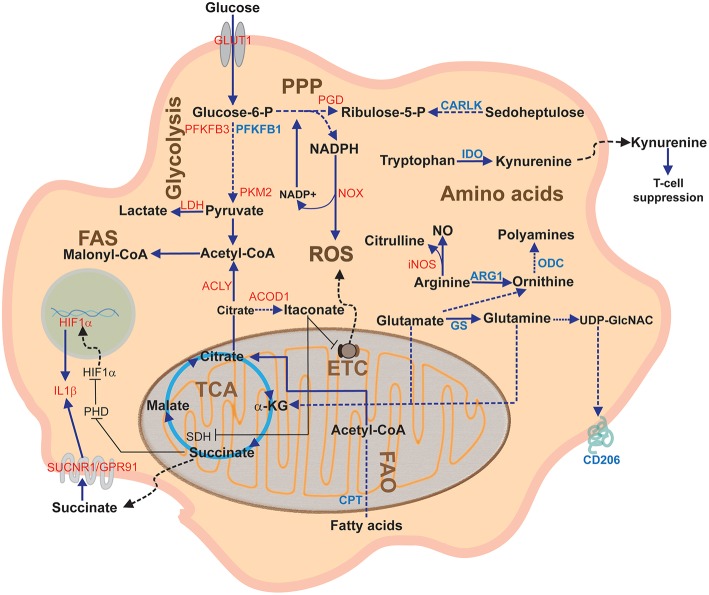
Overview of macrophage metabolic pathways. This diagram depicts the main macrophage pathways during classical and alternative polarization and their components. In red, proteins upregulated in pro-inflammatory (M1) activation; in blue, proteins upregulated during anti-inflammatory (M2) activation. α-KG, alpha-ketoglutarate; ACLY, ATP citrate lyase; ARG1, arginase1; CARLK, *car*bohydrate *k*inase-*l*ike protein; CPT, carnitine palmitoyl transferase; ETC; Electron Transport Chain; FAO Fatty acid oxidation; FAS, Fatty acid synthesis; GS, glutamine synthetase; GLUT1, glucose transporter 1; IDH, Isocitrate dehydrogenase; IDO, indoleamine dioxygenase; iNOS, inducible nitric oxide synthase; LDH, lactate dehydrogenase; NO, nitric oxide; NOX, NADPH oxidase; ODC, ornithine decarboxylase; PGD, phosphogluconate dehydrogenase; PHD, prolyl hydroxylase; PPP, Pentose phosphate pathway; PFKFB3, phosphofructokinase fructose 2,6-biphosphatase B3; PKM2, pyruvate kinase M2; ROS, Reactive Oxygen Species; SDH, Succinate dehydrogenase; SUCNR1, succinate receptor 1; TCA, Tricarboxylic acid cycle or Krebs cycle.

Because of its major role in controlling cell metabolism, citrate plays a crucial role in sustaining macrophage inflammatory response (Figure 3). M1 macrophages are characterized by accumulation of citrate due to two main transcriptional changes, such as downregulation of IDH (32) and upregulation of the mitochondrial citrate carrier CIC (71), leading to citrate withdrawal from mitochondria. CIC upregulation occurs in response to LPS, TNF-α, or IFN-γ stimulation. Both these events are responsible for the first interruption of the Krebs cycle and accumulation of citrate in the cytosol of M1 macrophages, which is crucial for NO, ROS, and prostaglandin E2 (PGE2) production (71, 78). Pharmacological or genetic targeting of CIC in human macrophages results in decreased levels of these inflammatory mediators (79), suggesting that citrate export supports fatty acid synthesis (FAS) on which PGE2 synthesis relies as well as the reduction of NADP^+^ to NADPH, necessary for NO and ROS production, by means of the activity of the malic enzyme. CIC acetylation is required to additionally boost mitochondrial citrate export in macrophages activated in conditions of glucose deprivation; in this manner, the NADPH demand, that cannot rely on PPP, may be met through the NADPH–producing conversion of citrate into 2-ketoglutarate, catalyzed by the cytosolic NADP^+^-dependent IDH (IDH1) (80).

Another crucial role of citrate is in providing, through conversion to acetyl-CoA, the acetyl moiety for the acetylation of proteins, which is known to regulate protein function at multiple levels (81). Protein acetylation requires the presence of acetyl-CoA in different cellular compartments and it relies on the activity of ATP citrate lyase (ACLY), which converts citrate in acetyl-CoA. Similarly to CIC, ACLY is upregulated in M1 macrophages (78) and its activity regulates the expression of many genes through histone acetylation (82). Although no specific studies address the role of ACLY in regulating epigenetic changes in M1 macrophages, many enzymes and proteins are known to be affected by acetylation (83), among which NF-κB (84), IL-6, and IL-10 (85, 86). In M2 macrophages ACLY is regulated by the Akt-mTORC1 axis, leading to histone acetylation and induction of some M2 genes (87). However, a recent study has shown that polarization of human macrophages toward an M2 phenotype does not require ACLY (88).

### Itaconate

Itaconate is produced from cis-aconitate in the Krebs cycle in classically activated macrophages (89, 90). This occurs through a strong upregulation of the enzyme aconitate decarboxylase 1 (ACOD1), originally called immune-responsive gene 1 protein (IRG1) (91). In this pathway, cis-aconitate is withdrawn from the Krebs cycle to produce this metabolite. Interestingly, upregulation of ACOD1 has been reported not only in cell lines and murine M1 macrophages, but also in septic patients (92).

The well-known anti-bacterial properties of itaconate rely on its ability to inhibit the bacterial isocitrate lyase and its bactericidal properties against gram-positive and gram-negative bacteria (93–95). In addition, itaconate may play a role in immunomodulation, suppression of inflammation and tolerance (96).

Itaconate was shown to inhibit SDH, leading to accumulation of succinate in LPS activated macrophages [(27–35); Figure 3], and this was associated to reduced mitochondrial respiration, ROS production, proinflammatory cytokine release, and inflammasome activation (96). The mechanism by which itaconate induces these metabolic and functional changes in macrophages was recently elucidated by Mills et al.: itaconate contributes to stabilize the levels of the anti-inflammatory transcription factor nuclear factor erythroid 2-related factor 2 (NRF2), which targets genes involved in anti-inflammatory and anti-oxidant response (97). Itaconate mediates a post-translational alkylating modification on –SH groups of Kelch-like ECH-associated protein 1 (KEAP1), causing its fast degradation. Since KEPA1 targets NRF2 for proteasomal degradation (98), its itaconate-mediated degradation allows NRF2 to translocate to the nucleus, leading to transcription of genes involved in protection against stress-induced cell death and oxidative stress. Concomitantly, NRF2 suppresses the expression of genes encoding IL-1β and IL-6 (99).

The chemical features of itaconate, particularly its electrophilicity, make the molecule reactive toward the cysteine groups of glutathione and proteins. It has been speculated that itaconate could trigger the electrophilic stress response (ESR), by modifying –SH residues of proteins and depauperating the cell from glutathione (100). It is then conceivable that other targets, besides the KEAP1–NRF2 axis, that are known to sense ESR, could be influenced by itaconate.

With respect to M2 macrophages, the role of itaconate has not been clearly elucidated. When M2 macrophage differentiation is impaired, IRG1 expression increases and itaconate accumulates in macrophages (101), probably as a compensatory effect. Furthermore, itaconate has been identified as a key player in the microRNA miR93, IRF9, IRG1 axis during macrophage polarization. In particular reduction of itaconate levels favors M2-like polarization in macrophages and this might be ascribed to the release of the SDH brake improving OXPHOS flux (102).

### Succinate

Succinate is an intermediate of the Krebs cycle produced from succinyl-CoA. It is the substrate of SDH, which is part of Complex II of the mitochondrial respiratory chain. SDH-mediated oxidation of succinate into fumarate is coupled to reduction of ubiquinone (UQ) to ubiquinol (UQH2). When high amounts of succinate are oxidized to fumarate in conditions of no ATP production, electrons flux in the opposite direction toward complex I, leading to reverse electron transport (RET). This associates to a significant release of ROS, which can activate HIF1α in M1 macrophages (65, 103).

In addition to its role as metabolic intermediate, succinate works as a signaling molecule in many ways. Succinate is transported into the cytosol by the activity of the dicarboxylate carrier (DIC), also known as solute carrier family 25 member 10 (SLC25A10) (72). Succinate influences HIF1α stability by inhibiting prolyl hydroxylases (PHDs), a class of αKG-dependent dioxygenases that regulate HIF1α stability in an oxygen-dependent manner, thus blocking HIF1α degradation in the presence of oxygen [(31); Figure 3].

High cytosolic succinate levels favor post-translational lysine succinylation on proteins, a process that profoundly modifies protein functions since it alters their charge and structure (104, 105). In the case of pyruvate kinase M2, succinylation promotes its translocation into the nucleus, where it interacts with HIF1α to boost IL-1β transcription (106). In sirtuin 5-deficient mice, pyruvate kinase M2 hyper-succinylation has been described as a strategy to sensitize mice to experimental colitis due to the increased IL-1β production (106).

Succinate exerts signaling roles also acting at the extracellular level. During inflammation, succinate is released by inflammatory macrophages and can accumulate into the extracellular milieu (107), as observed in murine ischemic or hypoxic tissue (108–110), inflammation of the central nervous system (111), as well as in biological fluids of rheumatoid arthritis patients (112). Once outside the cell, it can bind to the succinate receptor SUCNR1/GPR91, a G-protein–coupled cell surface sensor for extracellular succinate (113) expressed in many cell types, that is known to be activated in diabetic retinopathy (108), diabetic renal disease (114), hypertension (113, 115), and atherothrombosis (116). Interestingly, macrophages express GPR91 and, in response to inflammatory signals like LPS, they activate a GPR91-mediated signal transduction that sustains the proinflammatory phenotype and leads to IL-1β production [(117, 118); Figure 3]. This represents a novel mechanism by which succinate fuels inflammation in a autocrine manner to sustain and amplify the inflammatory response (118). Interestingly, in an *in vivo* model of experimental autoimmune encephalomyelitis (EAE), GPR91 expressed by transplanted neural stem cells exerted a protective role against neuroinflammation which was mainly due to their scavenging effects and reduction of the succinate levels in the cerebrospinal fluid (111).

## Amino Acid Metabolism in the Innate Immunity

Amino acid availability is essential to mount a proper immune response. During inflammatory or immune reactions, amino acid deficiency may result in defective immune cell migration, division, maturation, and completion of effector functions. Macrophage adaptation to rapidly changing nutrient sources implicates exploiting amino acid catabolism to sustain activation and maintenance of their immune activity. Amino acid availability controls several pathways governing macrophage responses, including mTOR signaling and NO production. Moreover, altered amino acid metabolism can influence macrophage responses by generating catabolites with immunomodulatory properties. Finally, the metabolic competition or cross talk between host immune cells and pathogens may affect the evolution of an infection.

Arginine represents the best example of how a strict metabolic regulation can drive opposite phenotypes, depending on which metabolic pathway is engaged (Figure 3). Under pro-inflammatory stimuli, such as LPS, TNF-α, or IFN-γ, iNOS (also known as NOS2) is overexpressed, channeling arginine catabolism toward NO and citrulline production. NO production is functional to boost macrophage anti-microbial activity: NO spontaneously reacts with oxygen or ROS to produce reactive nitrogen and oxygen intermediates that lead to the formation of a variety of antimicrobial species (119). Most importantly, NO prevents M1 to M2 repolarization, since the blockade of iNOS gives to M1 macrophages the ability to repolarize into M2, when exposed to IL-4 after LPS + IFN-γ treatment (120). On the other hand, citrulline produced by iNOS is used by argininosuccinate synthase 1 to produce argininosuccinate, which is promptly broken to recover arginine and sustain NO production (121). In contrast to M1 macrophages, anti-inflammatory M2 macrophages overexpress ARG1 that produces ornithine and urea from arginine catabolism. Ornithine is transformed by ornithine decarboxylase (ODC) to polyamines (putrescine, spermidine, and spermine) that control cell growth and are important for tissue repair. Interestingly, it has been recently reported that ODC limits M1 activation and macrophage anti-microbial activities by chromatin modification (122). Moreover, arginase competes with iNOS for arginine, and many pathogens exploit this by increasing expression of arginase and thus block NO production (123, 124). ARG1 activity in macrophages triggers an anti-inflammatory phenotype and reduces T-cell proliferation and cytokine production (117).

Although ARG1 and iNOS are competitively regulated by Th1 and Th2 cytokines and complex intracellular biochemical pathways, including negative feed-back loops and competition for the same substrate (125), simultaneous activation of ARG and NOS pathways occurs in myeloid cells licensed by the tumor (126). In tumor-infiltrating myeloid cells, L-Arg is metabolized by ARG1, ARG2, and iNOS. ARG and NOS co-activation within the same environment leads to production of several ROS and reactive nitrogen species (RNS) by the iNOS reductase domain at low L-Arg concentrations (127–131). Peroxynitrite produced by myeloid and tumor cells can nitrate tyrosine residues in the TCR and CD8 receptors, resulting in decreased recognition of peptide–MHC complexes (132) and T cell dysfunction (133); moreover, RNS can induce post-transcriptional modifications of chemokines and thus prevent intra-tumoral infiltration of antigen-specific T cells (134).

Tryptophan metabolism is a major mechanism of peripheral immune tolerance. In immune cells, the limiting step of tryptophan catabolism is mediated by indoleamine 2,3-dioxygenase (IDO) that converts tryptophan into kynurenine (Figure 3). Although IDO expression is induced by IFN-γ, TNF-α, or prostaglandins, macrophages are driven toward an M2 phenotype when IDO is overexpressed, and IDO silencing promotes a pro-inflammatory macrophage profile (135). Macrophages that express high levels of IDO may deplete extracellular tryptophan, thus affecting T-cell proliferation and functions (136, 137). TAMs, and sometimes tumor cells themselves, upregulate IDO and create an immunosuppressive microenvironment via at least two mechanisms: tryptophan depletion and accumulation of tryptophan catabolites, such as kynurenine, 3-hydroxyanthranilate, and quinolinate (137–139). From a mechanistic point of view, while tryptophan depletion inhibits rapid expansion of activated T cells, tryptophan-derived catabolites act as ligands of the aryl hydrocarbon receptors (AHR) (140). Kynurenine is a potent suppressor of T cell immunity: by stimulating AHR, it skews the differentiation of naive T cells toward FoxP3^+^ regulatory T cells (Tregs), whereas it suppresses T_h_17 cells differentiation (141).

In immune cells, glutamine is used for amino acid and nucleotide synthesis, NADPH and energy production, and many other biosynthetic pathways involved in cell proliferation and functions. Macrophages utilize glutamine at high rates and are dependent upon extracellular sources of the amino acid (76, 142). During macrophage activation, the different routes of glutamine consumption direct its role to promote either the M1 or M2 phenotype. Channeling of glutamine into the Krebs cycle is the main route to promote succinate synthesis in M1 macrophages, with the GABA shunt (a bypass of the TCA cycle in which glutamine is used for synthesis of glutamate, GABA, succinic semialdehyde, and eventually succinate) also playing a role (31). This is fundamental to stabilize HIF1α (33). On the other hand, glutamine metabolism drives M2 polarization by acting at multiple levels: (i) α-ketoglutarate generated from glutaminolysis is essential for M2 OXPHOS and FAO; (ii) α-ketoglutarate generated from glutaminolysis promotes an M2 phenotype by macrophage epigenetic reprogramming, involving demethylation of H3K27 on the promoters of M2-specific marker genes (143); (iii) α-ketoglutarate generated from glutaminolysis favors PHD activity and thus inhibits HIF1α expression; (iv) glutamine provides substrate for the UDP-GlcNAc synthesis (Figure 3). Indeed, the pathway for UDP-GlcNAc synthesis is upregulated in M2 macrophages and is essential for the glycosylation of different proteins expressed abundantly in M2 macrophages (32). Tracing experiments in M2 cells with ^13^C- and ^15^N-glutamine have shown that a third of all carbon in TCA metabolites and more than half of the nitrogen in UDP-GlcNAc derive from glutamine (32), providing further evidence for the essential role of glutamine metabolism in M2 differentiation of macrophages. Thus, M2 cells do not exclusively rely on glutamine uptake for their metabolism, but they induce glutamine synthesis from glutamate and ammonia via glutamine synthetase (GS). While GS is barely detectable in M1 macrophages, highly GS expression in M2 macrophages, particularly in response to IL-10, is fundamental for the acquisition of a M2-like phenotype (144). Indeed, GS inhibition skews IL-10 stimulated macrophages to a M1-like state, through a mechanism involving metabolic reprogramming (144, 145). Additionally, GS ablation in TAMs reduces M2 markers, such as ARG1 and CD206, and decreases tumor metastasis in mice (144, 145).

## Lipid Metabolism in Innate Immunity

Cellular lipid metabolism comprehends several key enzymatic processes that lead to the synthesis or the degradation of lipids (cholesterol, fatty acids, and phospholipids, Figure 2). As specialized phagocytic cells, macrophages are capable to uptake different forms of lipids such as LDL, VLDL, and oxidized lipoproteins from both engulfed dying cells and microenvironment via phagocytosis, macropinocytosis, and scavenger receptor-mediated pathways (146). After that, all ingested lipids are processed by acid lipases within the lysosomes, leading to the generation of free fatty acids and cholesterol (147). Free fatty acids are subsequently transported into mitochondria, where they are converted by the FAO pathway into different products that continuously replenish the TCA cycle with acetyl-coenzyme A, or the ETC through the generation of NADH and FADH2 (148).

On the other side, if metabolically required, FAS is induced through mTOR signaling within the cytosol. Notably, the FAS pathway permits the generation of lipids by using different precursors of TCA cycle, glycolysis and PPP pathway (149).

Transcriptional regulation of lipid metabolism is tightly controlled by sterol receptor element binding protein (SREBP) and liver X receptor (LXR). In macrophages, SREBP-1a and LXRα are highly expressed and regulate cytokine release and cell responses (150, 151). LPS treatment increases macrophage SREBP-1a activity via NF-κB, and macrophages deficient in SREBP-1a fail to produce IL-1β upon LPS stimulation (152), thus connecting lipid metabolism and inflammasome activation in M1 macrophages (153). In contrast, M2 macrophages are characterized by LXR activation, which regulates cholesterol homeostasis and lipid synthesis (154). Overexpression or activation of LXRα dampens M1 responses and inflammation by inhibiting the activity of NF-κB and AP-1 (155, 156).

It is clear that differential induction of FAS and FAO elicits macrophage polarization toward the M1 and M2 profiles, respectively (Figure 3). FAS represents an important pathway for energy production and prostaglandin biosynthesis in M1 cells; moreover, the accumulation of malonyl-CoA, product of the first step of FAS, can induce post-translational modifications (malonylation) that modulate responses of pro-inflammatory macrophages. On the other side, the main sources of fatty acids in M2 derive from uptake via the scavenger receptor-mediated pathway (157) and through lysosomal lipolysis mediated by lysosomal acid lipase, all these pathways being up-regulated upon IL-4 stimulation (147). M2 macrophages rely on fatty acid uptake and oxidation, which are supported by STAT6, the PPARγ (158) and its co-activator 1 (PGC1) (159). Indeed, M2 polarization could be prevented by inhibiting FAO, using a pharmacological approach targeting the carnitine palmitoyltransferase (CPT) system which mediates fatty acid translocation within mitochondria (160, 161). However, it has been recently reported that genetic ablation of CPT2 does not prevent macrophage polarization toward M2 profile upon IL-4 stimulation, both *in vitro* and *in vivo* (162). Interestingly, a recent work highlights the involvement of glutaminolysis-derived α-ketoglutarate as a positive metabolic regulator of FAO (163), thus suggesting further connections between different metabolic pathways.

## Macrophage Metabolism in Diseases

Alterations in macrophage polarization, function, and metabolic signature are present in various human diseases. In inflammatory diseases, such as sepsis, rheumatoid arthritis, and atherosclerosis, as well as in metabolic diseases, including obesity and diabetes, macrophages display prolonged or atypical M1 polarization; on the other hand, cancer growth is often associated with “M2-like” responses of TAMs (25, 164). A discussion of the role of macrophage metabolic adaptations in human diseases is not the focus of our review but here we briefly analyze some aspects of macrophage metabolic pathways in two conditions: obesity and cancer.

Among the immune cells that infiltrate obese adipose tissue, macrophages are functionally and numerically dominant (165). In the adipose tissue of lean mice, macrophages are 10–15% of cells, whereas they represent 45–60% of cells in the adipose tissue of obese animals (166). In addition to the difference in their numbers, adipose tissue macrophages in lean and obese animals exhibit distinct localizations and responses. Adipose tissue macrophages in lean animals have an alternatively activated (M2) phenotype, are anti-inflammatory and are uniformly dispersed throughout the adipose tissue, whereas adipose tissue macrophages of obese mice have a pro-inflammatory, classical (M1) phenotype and are primarily found in “crown-like” structures around dying adipocytes (167, 168). In lean adipose tissue, M2 macrophages have a crucial role in maintaining the insulin sensitivity of adipocytes via the secretion of interleukin-10 (IL-10) (158, 167), a regulatory cytokine that potentiates insulin signaling in adipocytes (165). By contrast, classically activated macrophages in obese adipose tissue secrete pro-inflammatory cytokines, which induce insulin resistance (165).

Fatty acids and TNF-α have been shown to induce M1 polarization in obesity (165) but also glucose, insulin, and obesity-induced hypoxia trigger macrophages toward a pro-inflammatory phenotype (169). Interestingly, in a study analyzing the metabolic signature in obese and normal-weight children, the serum concentration of glutamine was lower in obese children than in normal-weight ones (170). Moreover, a high ratio of glutamine to glutamate in the plasma is associated with a lower risk of diabetes mellitus (171). Because skeletal muscles participate in maintaining the concentration of glutamine in serum, the reduction in muscle mass in obese patients may account for reduced serum concentrations of glutamine (172). In addition, in the subcutaneous adipose tissues of obese patients there are higher concentrations of glutaminase and lower concentrations of glutamine synthase, compared with the lean subjects (173). Glutamine is known to promote M2 macrophage polarization (see above) and thus glutamine supplementation may represent a strategy to target macrophage polarization in obesity. Although a few studies have shown that therapeutic administration of glutamine is beneficial in obesity and diabetes, the direct effects of this metabolic targeting on macrophages is not proven (172).

As already discussed, the succinate receptor SUCNR1/GPR91 is known to play a role in several diseases including diabetic retinopathy (108), diabetic renal disease (114), hypertension (113, 115), atherothrombosis (116), neuroinflammation (111), rheumatoid arthritis (114), and metabolic dysfunctions (174). Interestingly, the concentration of succinate in plasma is higher in patients with type 2 diabetes than in non-diabetic individuals (174) and it is significantly associated with the body mass index (BMI) (175). Thus, targeting succinate and its receptors may represent an interesting therapeutic strategy to modulate macrophage responses and inflammation in several pathological contexts.

It is highly probable that the metabolic signature of TAMs depends on the surrounding microenvironment and may thus be different in different tissues. In general terms, and on the basis of the recent publications, TAMs seem to depend on glycolysis for their metabolic needs and produce lactate at high concentration (79, 176). Lactate (177), in turn, induces VEGF and ARG1, thus promoting a pro-angiogenic signature, and potentiates glycolysis by activating the Akt/mTOR pathway. However, when cancer grows, hypoxia induces in TAMs up-regulation of REDD1 (regulated in development and in DNA damage response 1), which inhibits mTOR, and thus inhibits glucose uptake by macrophages and glycolysis. This is associated with an increased angiogenic response and formation of aberrant leaky vessels due to enhanced glucose availability for endothelial cells (178).

As mentioned above, TAM-produced lactate induces ARG1, which depletes arginine and is directly involved in TAM-induced immunosuppression. On the other hand, highly overexpressed cyclooxygenase in TAMs induces IDO expression, which depletes tryptophan thus suppressing T cell responses. Therefore, macrophage functions in the tumor microenvironment are regulated by a complex and interconnected reprogramming involving glucose, amino acid, and lipid metabolism.

## Conclusions

Macrophage metabolic adaptations have been deeply analyzed during the last years and have emerged as critical factors regulating a variety of cell responses. Pathogen or inflammatory signals drive macrophage differentiation toward the acquisition of new functions by rapidly modulating the expression of key genes. Associated to this program is a remodeling of the metabolic pathways that sustains, from an energetic, biosynthetic, and regulatory point of view, its execution (Figure 3). Information from studies on inflammation-linked diseases is teaching us that pathological immune response might be underpinned by aberrant metabolic rewiring (145, 179, 180). Several metabolic products play important roles as signaling mediators, affecting not only macrophages but also neighboring cells, thus representing interesting targets for therapeutic strategies (181). To this aim, it is fundamental to understand the interplay between metabolism and immunity by dissecting the different metabolic reactions important for the acquisition of specific functions. On the other hand, it is becoming evident that the different metabolic pathways are strongly interconnected and that positive and negative feedback loops are involved in amplification or dampening of immune responses. Thus, as growing literature defines metabolic processes and pathways in macrophages, the ultimate goal is an integrated view of the metabolic networks regulating inflammation, immunity, and tissue responses to homeostasis perturbations.

## Author Contributions

FM, RS-R, TS, and AC wrote the manuscript. AV and AC reviewed the manuscript.

### Conflict of Interest Statement

The authors declare that the research was conducted in the absence of any commercial or financial relationships that could be construed as a potential conflict of interest.
